# Causal relationships between rheumatoid arthritis and neurodegenerative diseases: a two-sample univariable and multivariable Mendelian randomization study

**DOI:** 10.3389/fmed.2024.1439344

**Published:** 2024-08-13

**Authors:** Xingyu Chen, Li Cai, Weibing Fan, Qian Yang, Xinfa Mao, Liping Yao

**Affiliations:** ^1^Department of Neurology, Hunan Provincial People's Hospital, The First Affiliated Hospital of Hunan Normal University, Changsha, China; ^2^Department of Neurology, The Third Hospital of Changsha, Changsha, China

**Keywords:** rheumatoid arthritis, Alzheimer's disease, Parkinson's disease, amyotrophic lateral sclerosis, Mendelian randomization

## Abstract

**Background:**

Observational research has highlighted a potential relationship between rheumatoid arthritis (RA) and neurodegenerative diseases (NDs). However, the confirmation of a causal connection is impeded by the inherent limitations of such studies, including vulnerability to confounding factors and the possibility of reverse causality. This study employs a two-sample Mendelian randomization (MR) approach to assess the causal impact of RA on three NDs, including Alzheimer's disease (AD), Parkinson's disease (PD), and amyotrophic lateral sclerosis (ALS).

**Methods:**

We aggregated data from genome-wide association studies (GWASs) targeting RA or NDs within populations of European descent. Single nucleotide polymorphisms (SNPs) with robust associations to RA were identified as instrumental variables (IVs). To estimate the association between RA and AD, PD, and ALS, we utilized the inverse variance weighted (IVW) method in our univariable MR (UVMR) analysis. Validation of the IVW results ensued through supplementary analyses using MR-Egger and weighted median methods. The multivariable MR (MVMR) analysis was conducted, adjusting for body mass index (BMI), alcohol drinking, and type 2 diabetes mellitus (T2DM).

**Results:**

The UVMR analysis, based on the IVW method, revealed a significantly positive causal association between RA and late-onset (LO) AD (OR [95% CI] = 1.084 [1.020–1.153]; *p* = 9.980 × 10^−3^), while suggesting a possible inverse relationship with PD (OR [95% CI] = 0.727 [0.563–0.938]; *p* = 0.014). Our study did not detect any causal connections between RA and early-onset (EO) AD, atypical or mixed (AM) AD, and ALS (all *p* > 0.05). The MVMR analysis results indicated that after adjusting for alcohol drinking, RA remains a risk factor for LOAD (OR [95% CI] = 1.094 [1.024–1.169]; *p* = 0.008). However, MVMR analysis revealed no causal connections between RA and PD after adjustments for BMI, alcohol drinking, or T2DM (all *p* > 0.05). Sensitivity analyses showed no evidence of heterogeneity and horizontal pleiotropy.

**Conclusions:**

This research provides genetic evidence indicating that RA potentially causes an increased risk of developing LOAD and PD. Such a revelation underscores the importance for individuals suffering from RA to be vigilant about the potential emergence of LOAD and PD. Ongoing monitoring and prompt detection are essential for successfully managing and intervening in this possible risk.

## 1 Introduction

Neurodegenerative diseases (NDs) represent a diverse and complex category of diseases marked by the progressive loss of neurons and degeneration across various sectors of the nervous system, exhibiting an escalating incidence (1). NDs are increasingly becoming a prevalent source of both morbidity and mortality, especially among the elderly. Among these, Alzheimer's disease (AD), Parkinson's disease (PD), and amyotrophic lateral sclerosis (ALS) stand out as significant NDs (2). Currently, AD impacts an estimated 35 million individuals worldwide, with projections suggesting a tripling of this figure by 2060 (3, 4). PD is observed in approximately 1% of those over the age of 65, with predictions indicating a fourfold increase by 2040 (5, 6). ALS has a global prevalence of about 4.42 per 100,000 individuals, with both prevalence and incidence rates expected to rise with the aging population (7, 8). Despite extensive research conducted on these major NDs, their pathophysiological mechanisms remain largely uncharted. The lack of clarity regarding their pathogenesis means that, to date, no effective cures have been identified. Consequently, NDs continue to place significant health, societal, and financial strains on communities across the globe.

Unraveling the complex mechanisms that drive disease is a cornerstone objective in contemporary medical science. The emergence of NDs typically stems from neurologic malfunctions and the demise of brain cells (9). However, the origins of NDs are multifaceted, with numerous critical elements contributing to their development (10). Presently, there is strong evidence to suggest that both inflammatory processes and immune responses are significant in the development of NDs (11, 12), and there is documented comorbidity with autoimmune conditions (13, 14). Rheumatoid arthritis (RA), the most prevalent autoimmune disease, is characterized by excessive inflammatory and immune reactions (15). RA primarily manifests with symptoms of joint rigidity, swelling, and reduced mobility, but it can also involve extra-articular organs including the eyes, lungs, skin, and the central nervous system (16). Epidemiological investigations into the association between RA and NDs have yielded conflicting outcomes. Conventionally, RA has been correlated with a heightened risk of PD in East Asian populations (17), attributed to heightened inflammatory and immune activity (18). Recent observational research, however, has identified an inverse association, indicating a lower risk of PD among individuals with RA within the European population, a discovery that contrasts with previous findings (19). Moreover, observational study in East Asia have demonstrated an increased incidence of AD among individuals with RA, in comparison to those without the condition (20). Simultaneously, research conducted in Europe suggested that people with arthritic conditions, especially RA, might encounter cognitive decline in their later years (21). Conversely, investigation in the United States has proposed that RA may impart a protective effect against AD, noting a reduced occurrence of AD among those with RA (22). Furthermore, epidemiological research revealed that that having certain autoimmune diseases, notably RA, does not correlate with a heightened risk of ALS (23).

However, the reliability of these observational studies is compromised by the potential for confounding variables, such as the administration of nonsteroidal anti-inflammatory drugs (NSAIDs) (19). The presence of various confounding elements in these studies often leaves the true causal link between RA and NDs ambiguous. The challenge in drawing causal conclusions from observational research lies in the vulnerability to biases, including reverse causation and the presence of confounders (24), which dilute our comprehension of the direct connection between RA and NDs. Mendelian randomization (MR) represents a novel analytical approach designed to investigate the causative links between exposures and outcomes (25). In MR analyses, genetic variants with a known association to the exposure of interest are leveraged as instrumental variables (IVs), thereby providing an estimation of causal effects. The advantage of genetic variants lies in their imperviousness to modification by external environmental or behavioral factors, representing a stable exposure variable over time. Through the application of MR, it is possible to circumvent the confounding (non-genetic components such as nutrition, lifestyle, environmental exposures, etc.) (26, 27) and reverse causation that often beset observational studies (28). In present study, we performed a two-sample univariable and multivariable MR analysis to explore the potential causative associations between RA and the incidence of three NDs (AD, PD, and ALS), aiming to provide novel possibilities for future therapeutic approaches.

## 2 Methods

### 2.1 Study design

**Figure 1:**
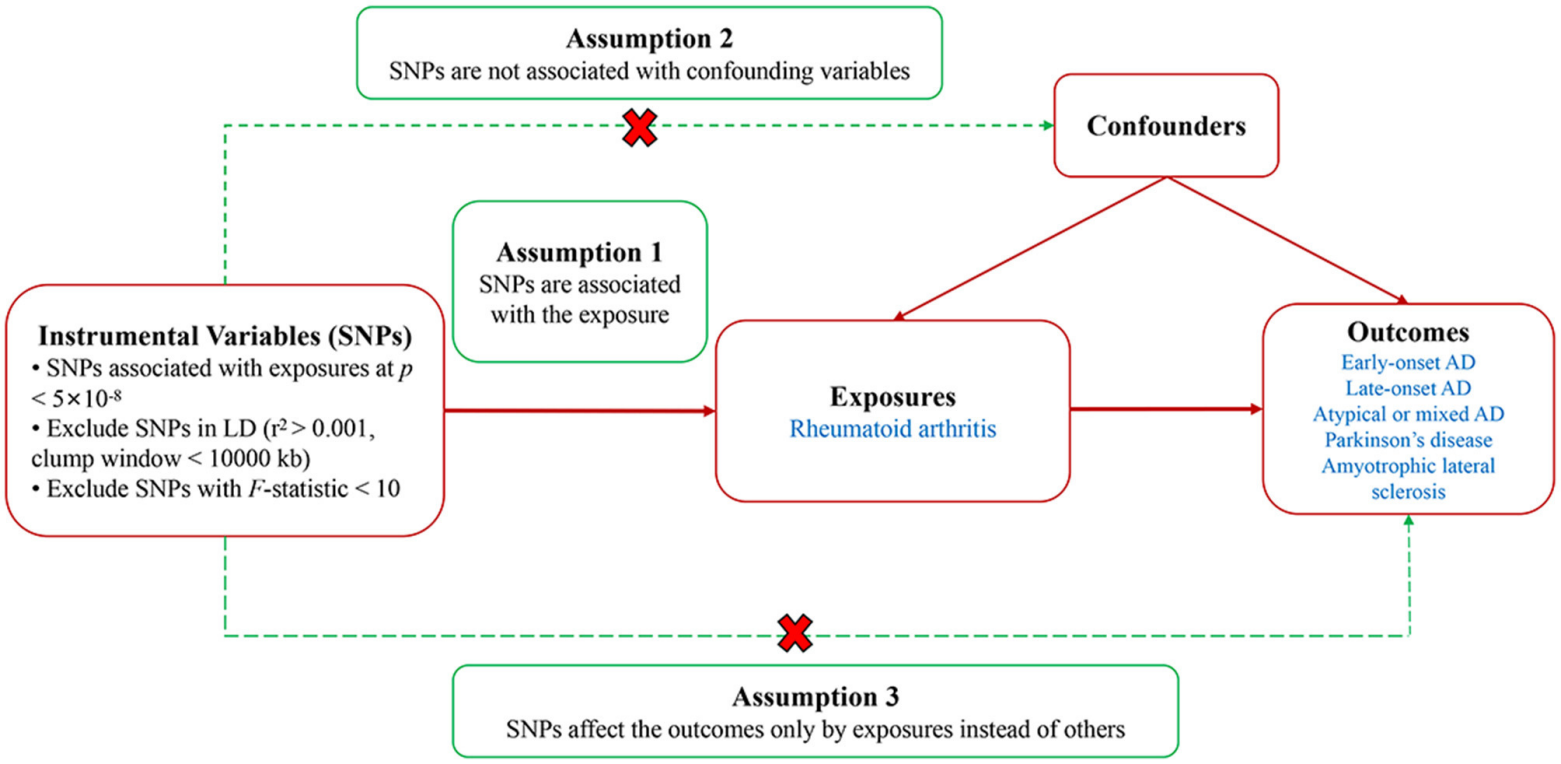
Study design diagram and three assumptions of Mendelian randomization. SNPs, single nucleotide polymorphisms; LD, linkage disequilibrium; AD, Alzheimer's disease.

Our investigation employed a two-sample univariable and multivariable MR technique to thoroughly evaluate the causal links between RA and AD, PD, and ALS. IVs, based on genetic variants-specifically single nucleotide polymorphisms (SNPs)-that have a strong correlation with RA, were employed in MR analysis. The validity of our MR methodology relied on meeting three fundamental criteria: (1) A robust association between the IVs and the exposure variable is necessary; (2) The selected SNPs must not be related to any confounding variables; (3) The IVs' influence on the outcomes was required to be mediated exclusively through the exposure pathway (29). Figure 1 encapsulated a schematic representation of our MR analytical process. Our methodological execution conformed meticulously to the STROBE-MR guidelines, ensuring the precision of our reported findings (30). Since the data for our analysis was sourced from pre-existing studies that had already secured ethical clearance, the requisition for additional ethical approval and informed consent was deemed unnecessary for this study.

### 2.2 Data sources

Genetic associations with RA were analyzed using data from the IEU Open genome-wide association study (GWAS) database (https://gwas.mrcieu.ac.uk/), encompassing a cohort of 417,256 participants. This dataset included 8,255 RA patients and 409,001 control subjects (31). The FinnGen consortium (https://r9.finngen.fi/), which pools genetic information from individuals of European descent who have provided informed consent, contributed with the genetic datasets for AD. This consortium integrates genetic and health data from the Finnish Biobank and National Health Registry, recording 1,314 instances of early-onset (EO) AD, 6,489 of late-onset (LO) AD, and 2,044 of atypical or mixed (AM) AD, with a comparison group of 170,429 individuals. For PD, we leveraged genetic summary data from the UK Biobank, which offers publicly accessible summary statistics for a cohort of 456,348 individuals of European lineage, including 294 PD cases and 456,054 controls (32). Furthermore, genetic information pertaining to ALS was sourced from the research conducted by Nicolas et al., which included data on 20,806 ALS patients and 59,804 control subjects (33).

Our research entailed the utilization of the PhenoScanner database (http://www.phenoscanner.medschl.cam.ac.uk/) to discern potential confounders, including body mass index (BMI), alcohol drinking, and type 2 diabetes mellitus (T2DM), as indicated by earlier studies (34, 35). We extracted summary data for these traits from research utilizing the UK Biobank, which included 407,609 British-ancestry participants for BMI analysis (36), 232,585 individuals of European descent for examining alcohol drinking (32), and 468,298 European-ancestry participants for T2DM analysis (37). Comprehensive details of these GWAS datasets were delineated in Table 1.

**Table 1:** The GWAS data source details in our study.

**Phenotype**	**Cases**	**Controls**	**Sample size**	**nSNP**	**Ethnicity**	**PMID**	**Data source**
**Exposures**							
RA	8,255	409,001	417,256	24,175,266	European	34594039	IEU Open GWAS
**Adjustments**							
BMI	407,609 British ancestry individuals			10,783,680	European	34017140	GWAS catalog
Alcohol drinking	232,585 European ancestry individuals			11,831,135	European	34737426	GWAS catalog
T2DM	468,298 European ancestry individuals			12,004,440	European	29892013	GWAS catalog
**Outcomes**							
AD (EO)	1,314	170,429	171,743	20,156,258	European	NA	FinnGen
AD (LO)	6,489	170,429	176,918	20,157,421	European	NA	FinnGen
AD (AM)	2,044	170,429	172,473	20,156,474	European	NA	FinnGen
PD	294	456,054	456,348	11,831,294	European	34737426	GWAS catalog
ALS	20,806	59,804	80,610	9,481,886	European	29566793	GWAS catalog

RA, Rheumatoid arthritis; BMI, body mass index; T2DM, type 2 diabetes mellitus; AD, Alzheimer's disease; EO, Early onset; LO, Late onset; AM, Atypical or mixed; PD, Parkinson's disease; ALS, Amyotrophic lateral sclerosis; NA, not available.

### 2.3 Instrumental variable selection

In the process of selecting genetic variants relevant to the exposure of RA, we followed a standardized protocol. The criterion for statistical significance was established at a *p* < 5 × 10^−8^ for RA exposure. To identify independent IVs, we applied linkage disequilibrium (LD) clumping techniques, setting an r^2^ threshold of 0.001 and a clumping window of 10,000 kb, based on LD information from the 1,000 Genomes Project. Subsequently, the variants with the lowest *p*-values were selected as the independent IVs (38). Furthermore, we evaluated SNPs for their association with RA by calculating the *F*-statistic, considering IVs with an *F*-value >10 to be robust (39). We then consulted the GWAS catalog (https://www.ebi.ac.uk/gwas/) to check for any associations between our selected SNPs and known confounders. Any SNPs that exhibited an association with the exposure and a direct link to the outcomes, with a *p*-value below 5 × 10^−8^, were excluded. Ultimately, we integrated the data from the exposure and outcomes, carefully excluding any palindromic sequences to guarantee the uniformity of allele effects.

### 2.4 MR analysis

To clarify the causal association between RA and AD, PD, and ALS, we adopted the inverse variance weighted (IVW) technique as our primary tool for univariable MR (UVMR) analysis. This method computes weighted summary effects in relation to the inverse of the variance, presuming all IVs are reliable. The IVW technique consolidates Wald ratios from individual SNPs, resulting in a comprehensive causal estimate (40). To corroborate the robustness of our results and to uncover any potential pleiotropic effects, we undertook additional analyses using MR-Egger regression and the weighted median method. MR-Egger regression, through its intercept, can identify pleiotropic influences, enabling adjustments in causal estimates, albeit potentially reducing statistical power (41). The weighted median method integrates information from multiple genetic variants, generating a robust causal estimate (42). We further refined our analysis using multivariable MR (MVMR) method, incorporating factors such as BMI, alcohol drinking, and T2DM to account for potential confounding variables. This included the use of multivariable IVW, multivariable MR-Egger, and multivariable median methods (43). Our findings attained statistical significance with *p* < 0.01 (0.05/5), following the application of the Bonferroni correction to adjust for multiple testing. A *p*-value between 0.01 and 0.05 was considered indicative of potential statistical significance.

### 2.5 Sensitivity analysis

The Cochran's Q test was applied to assess the heterogeneity among the genetic variance estimates. A *p* < 0.05 from the Cochran's Q test indicated the necessity for a random-effects model in subsequent MR analysis. In contrast, a *p*-value above this cutoff suggested that a fixed-effects model was more suitable (44). The MR-Egger intercept was used to evaluate the presence of horizontal pleiotropy, with *p*-values exceeding 0.05 signifying an absence of pleiotropy (41). To identify and mitigate the impact of outliers on causal inferences, the study incorporated the MR pleiotropy residual sum and outlier (MR-PRESSO) technique (45). Additionally, a leave-one-out strategy was employed to identify IVs that could potentially influence the estimation of causal effects, by sequentially excluding each SNP and observing the impact on the remaining set. The associations between RA and AD, PD, and ALS were illustrated through scatter plots and forest plots. To confirm the robustness of our findings, funnel plot analysis was also undertaken. All statistical analyses were carried out using R software 4.3.1, employing the “TwoSampleMR” and “MendelianRandomization” packages.

## 3 Results

### 3.1 Selection of instrumental variables

In our initial evaluation, we identified 25 candidate SNPs to serve as IVs for RA. However, rs6679677 was eliminated from consideration due to its pronounced association with type 1 diabetes (*p* < 8 × 10^−24^), posing a risk of confounding. Similarly, rs34536443 was eliminated because of its significant linkage to both type 1 and type 2 diabetes (*p* < 2 × 10^−11^). Additionally, rs3093017 was excluded due to its palindromic nature and intermediate allele frequencies. Following the stringent selection process, we identified 19 SNPs for AD, 21 for PD, and 21 for ALS to be utilized as IVs in the MR analyses. The robustness of these chosen SNPs was validated by *F*-statistics exceeding 10, indicating the absence of weak instrument bias. Detailed information on these SNPs can be found in Supplementary Tables 1–6.

### 3.2 UVMR analysis of RA on AD, PD, and ALS

**Figure 2:**
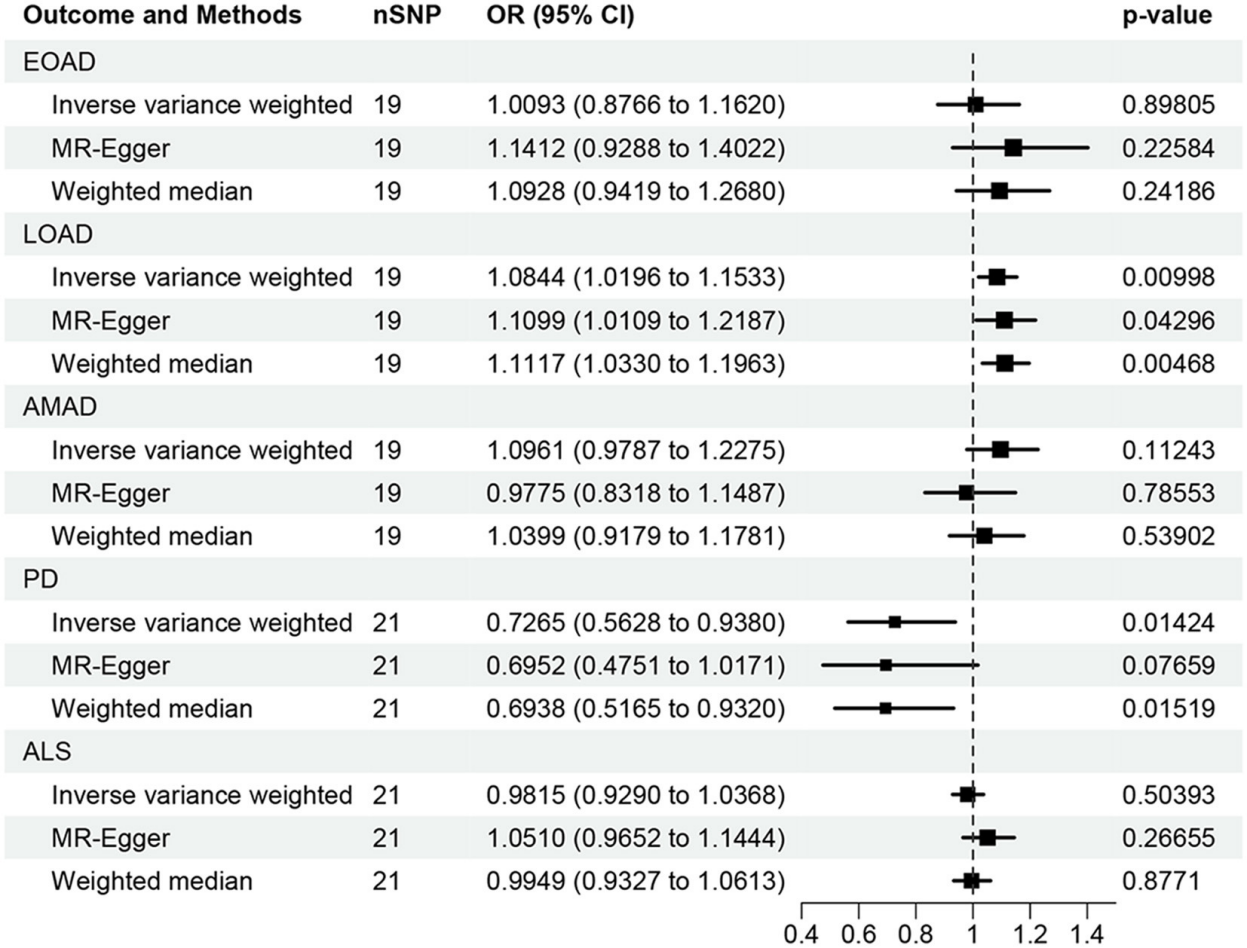
MR analysis of the causal effect of RA on AD, PD, and ALS. AD, Alzheimer's disease; EO, Early-onset; LO, Late-onset; AM, Atypical or mixed; PD, Parkinson's disease; ALS, Amyotrophic lateral sclerosis.

**Figure 3:**
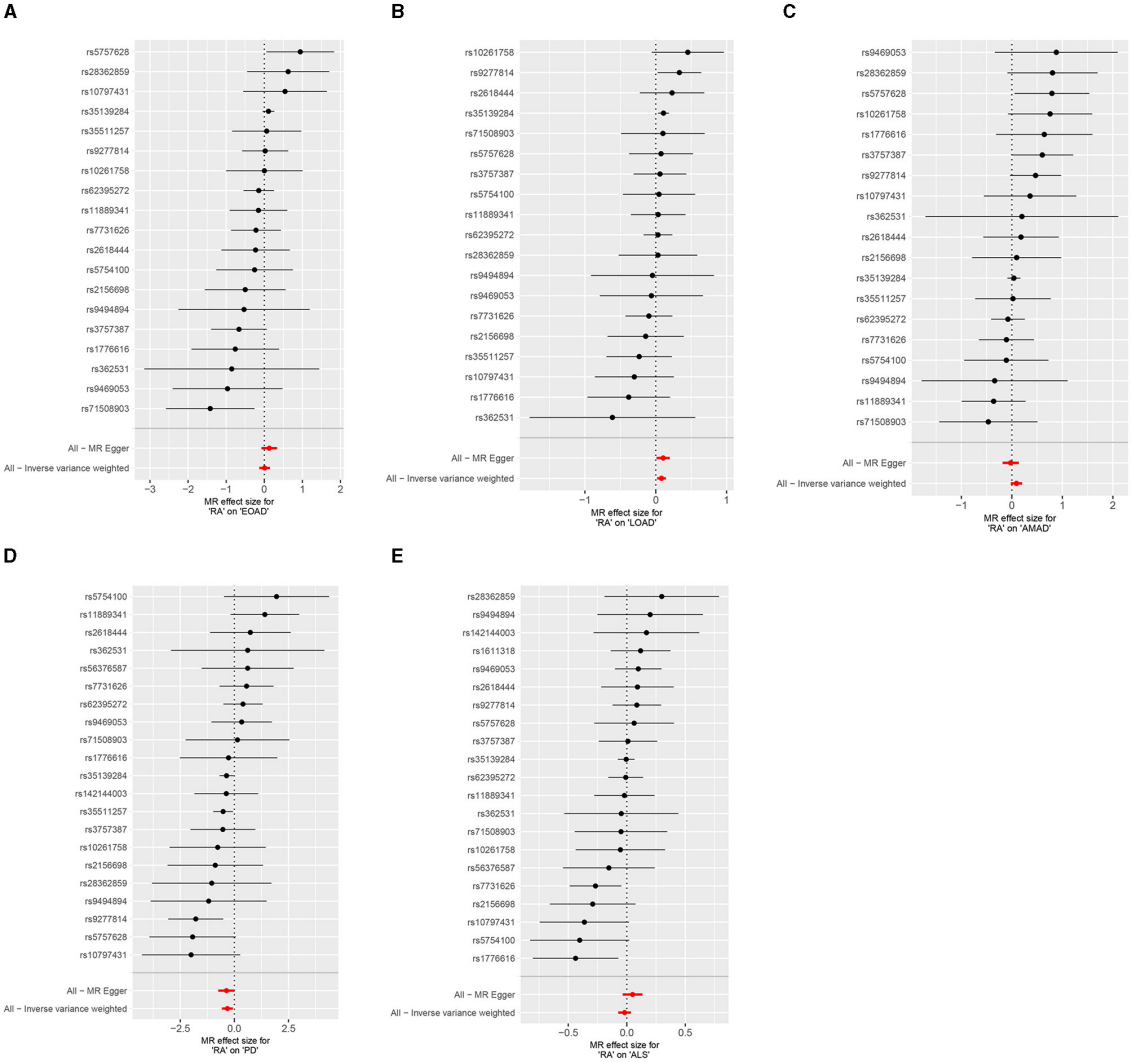
Forest plots of RA on AD, PD, and ALS. **(A)** Early-onset Alzheimer's disease; **(B)** Late-onset Alzheimer's disease; **(C)** Atypical or mixed Alzheimer's disease; **(D)** Parkinson's disease; **(E)** Amyotrophic lateral sclerosis.

**Figure 4:**
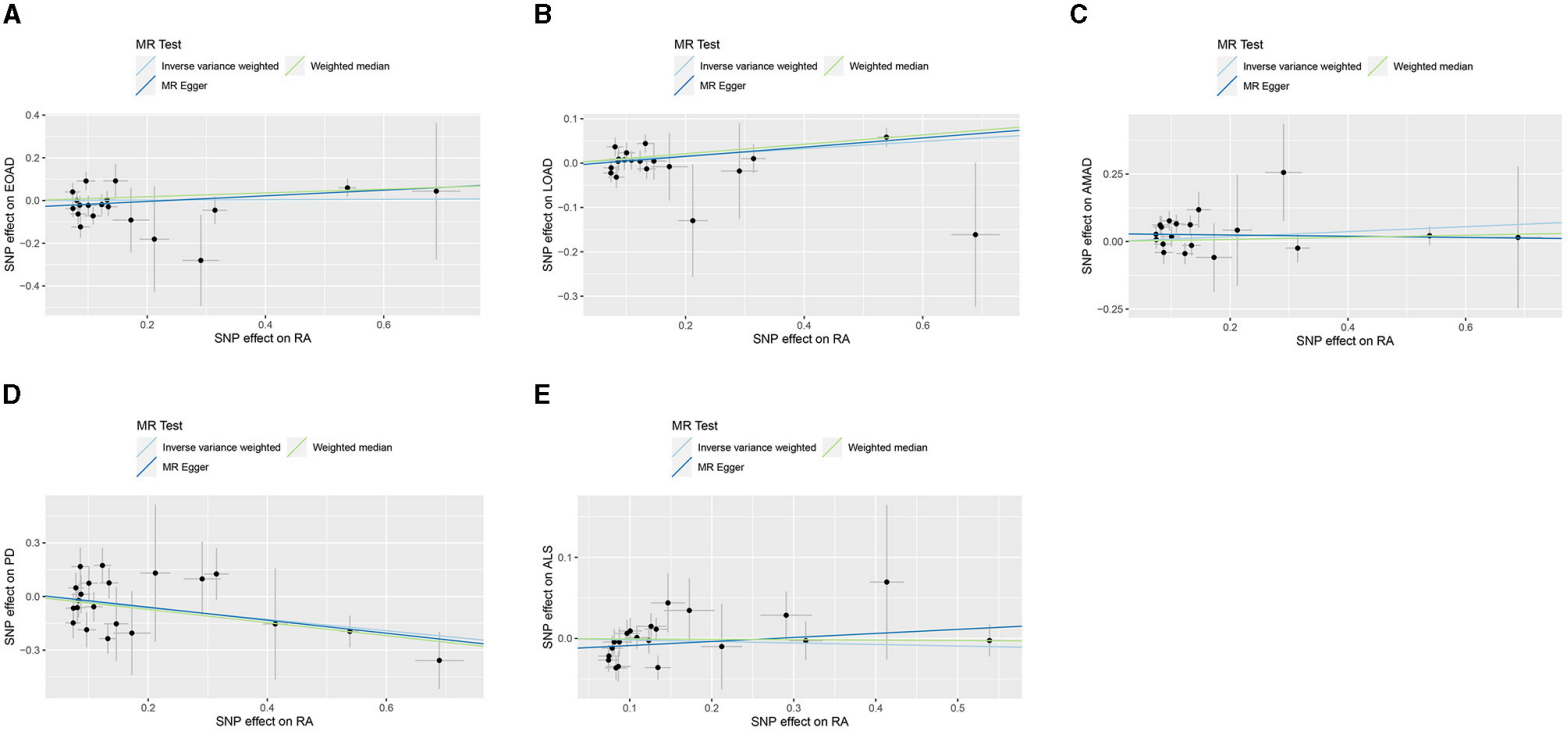
MR analysis scatter plots of RA on AD, PD, and ALS. **(A)** Early-onset Alzheimer's disease; **(B)** Late-onset Alzheimer's disease; **(C)** Atypical or mixed Alzheimer's disease; **(D)** Parkinson's disease; **(E)** Amyotrophic lateral sclerosis.

In the present research, we probed the relationships between RA and AD, PD, and ALS, with the results depicted in Figure 2. Through the application of the IVW method, we identified a significant positive association between RA and LOAD (OR [95% CI] = 1.084 [1.020–1.153]; *p* = 9.980 × 10^−3^). This association was further substantiated by subsequent MR analyses using both MR-Egger (OR [95% CI] = 1.110 [1.011–1.219]; *p* = 0.043) and the weighted median approach (OR [95% CI] = 1.112 [1.033–1.196]; *p* = 0.005), thereby strengthening the evidence of a positive causal connection. In contrast, the IVW method suggested a possible protective effect of RA against developing PD (OR [95% CI] = 0.727 [0.563–0.938]; *p* = 0.014), which was also supported by results from the weighted median approach (OR [95% CI] = 0.694 [0.517–0.932]; *p* = 0.015). However, our study failed to uncover any significant relationship between RA and EOAD (OR [95% CI] = 1.009 [0.877–1.162]; *p* = 0.898), AMAD (OR [95% CI] = 1.096 [0.979–1.228]; *p* = 0.112), or ALS (OR [95% CI] = 0.982 [0.929–1.037]; *p* = 0.504), with neither the MR-Egger nor the weighted median method showing significant results (all *p* > 0.05). The forest plots in Figure 3 presented the estimated causal effects between RA and these NDs. Additionally, Figure 4 showed scatter plots with MR intercepts close to zero, indicating a negligible influence of horizontal pleiotropy in the analyses conducted.

### 3.3 MVMR analysis of RA on AD, PD and ALS

In the MVMR analysis, the multivariable IVW method did not reveal any significant associations between RA and EOAD, AMAD, and ALS after adjustments for BMI, alcohol drinking, or T2DM (all *p* > 0.05). These non-significant findings were also obtained from multivariable MR-Egger and median methods (all *p* > 0.05). However, the MVMR analysis demonstrated a significant positive relationship between RA and LOAD using multivariable IVW (OR [95%CI] = 1.094 [1.024–1.169]; *p* = 0.008) and MR–Egger (OR [95%CI] = 1.126 [1.039–1.221]; *p* = 0.004) methods after adjusting for alcohol drinking. In contrast, after adjustment for alcohol drinking, MVMR analysis suggested that RA could potentially decrease the risk of PD based on multivariable MR–Egger (OR [95%CI] = 0.710 [0.541–0.931]; *p* = 0.013) and MVMR median (OR [95%CI] = 0.700 [0.512–0.958]; *p* = 0.026) methods; however, this potential protective relationship was not corroborated by the multivariable IVW method (OR [95%CI] = 0.799 [0.635–1.004]; *p* = 0.054). When adjustments for BMI or T2DM were made, no correlation was discovered between RA and either LOAD or PD (all *p* > 0.05) (Table 2).

**Table 2:** Multivariable Mendelian randomization (MVMR) analysis of rheumatoid arthritis with neurodegenerative diseases.

**Outcomes**	**Adjustments**	**Methods**	**nSNP**	**OR**	**95% CI**	***p* value**	**Egger Intercept**	***p* value**
AD (EO)	BMI	Inverse variance weighted	435	0.950	0.796–1.133	0.570	−0.002	0.465
		MR-Egger	435	0.996	0.802–1.239	0.972		
		MVMR median	435	0.920	0.742–1.141	0.448		
	Alcohol drinking	Inverse variance weighted	39	1.017	0.892–1.158	0.799	−0.010	0.432
		MR-Egger	39	1.057	0.899–1.242	0.505		
		MVMR median	39	0.930	0.775–1.116	0.435		
	T2DM	Inverse variance weighted	93	0.975	0.817–1.164	0.782	0.012	0.095
		MR-Egger	93	0.875	0.703–1.087	0.226		
		MVMR median	93	0.931	0.735–1.182	0.560		
AD (LO)	BMI	Inverse variance weighted	435	1.035	0.921–1.162	0.562	−0.002	0.448
		MR-Egger	435	1.068	0.927–1.232	0.362		
		MVMR median	435	1.108	0.984–1.250	0.090		
	Alcohol drinking	Inverse variance weighted	39	1.094	1.024–1.169	0.008	−0.007	0.224
		MR-Egger	39	1.126	1.039–1.221	0.004		
		MVMR median	39	1.075	0.996–1.160	0.065		
	T2DM	Inverse variance weighted	93	1.089	1.000–1.186	0.051	0.003	0.429
		MR-Egger	93	1.062	0.955–1.181	0.268		
		MVMR median	93	1.070	0.944–1.214	0.291		
AD (AM)	BMI	Inverse variance weighted	435	1.035	0.891–1.201	0.657	0.002	0.397
		MR-Egger	435	0.988	0.822–1.186	0.895		
		MVMR median	435	1.096	0.908–1.324	0.340		
	Alcohol drinking	Inverse variance weighted	39	1.058	0.960–1.164	0.260	0.004	0.683
		MR-Egger	39	1.042	0.924–1.174	0.503		
		MVMR median	39	1.041	0.920–1.177	0.525		
	T2DM	Inverse variance weighted	93	1.114	0.971–1.278	0.123	0.005	0.311
		MR-Egger	93	1.058	0.893–1.254	0.516		
		MVMR median	93	1.112	0.906–1.365	0.311		
PD	BMI	Inverse variance weighted	466	0.964	0.736–1.260	0.785	0.005	0.362
		MR-Egger	466	0.895	0.654–1.223	0.484		
		MVMR median	466	1.390	0.887–2.177	0.151		
	Alcohol drinking	Inverse variance weighted	41	0.799	0.635–1.004	0.054	0.033	0.130
		MR-Egger	41	0.710	0.541–0.931	0.013		
		MVMR median	41	0.700	0.512–0.958	0.026		
	T2DM	Inverse variance weighted	93	0.785	0.598–1.031	0.083	−0.009	0.491
		MR-Egger	93	0.831	0.605–1.143	0.257		
		MVMR median	93	0.780	0.491–1.239	0.291		
ALS	BMI	Inverse variance weighted	482	1.005	0.947–1.066	0.867	< 0.001	0.803
		MR-Egger	482	1.011	0.938–1.090	0.775		
		MVMR median	482	0.972	0.897–1.054	0.492		
	Alcohol drinking	Inverse variance weighted	40	0.980	0.930–1.035	0.477	−0.003	0.438
		MR-Egger	40	0.997	0.931–1.068	0.936		
		MVMR median	40	0.994	0.927–1.066	0.871		
	T2DM	Inverse variance weighted	92	0.977	0.923–1.036	0.440	−0.002	0.313
		MR-Egger	92	0.999	0.931–1.073	0.980		
		MVMR median	92	0.981	0.898–1.071	0.668		

AD, Alzheimer's disease; EO, Early onset; BMI, body mass index; T2DM, type 2 diabetes mellitus; LO, Late onset; AM, Atypical or mixed; PD, Parkinson's disease; ALS, Amyotrophic lateral sclerosis.

### 3.4 Sensitivity analyses

**Figure 5:**
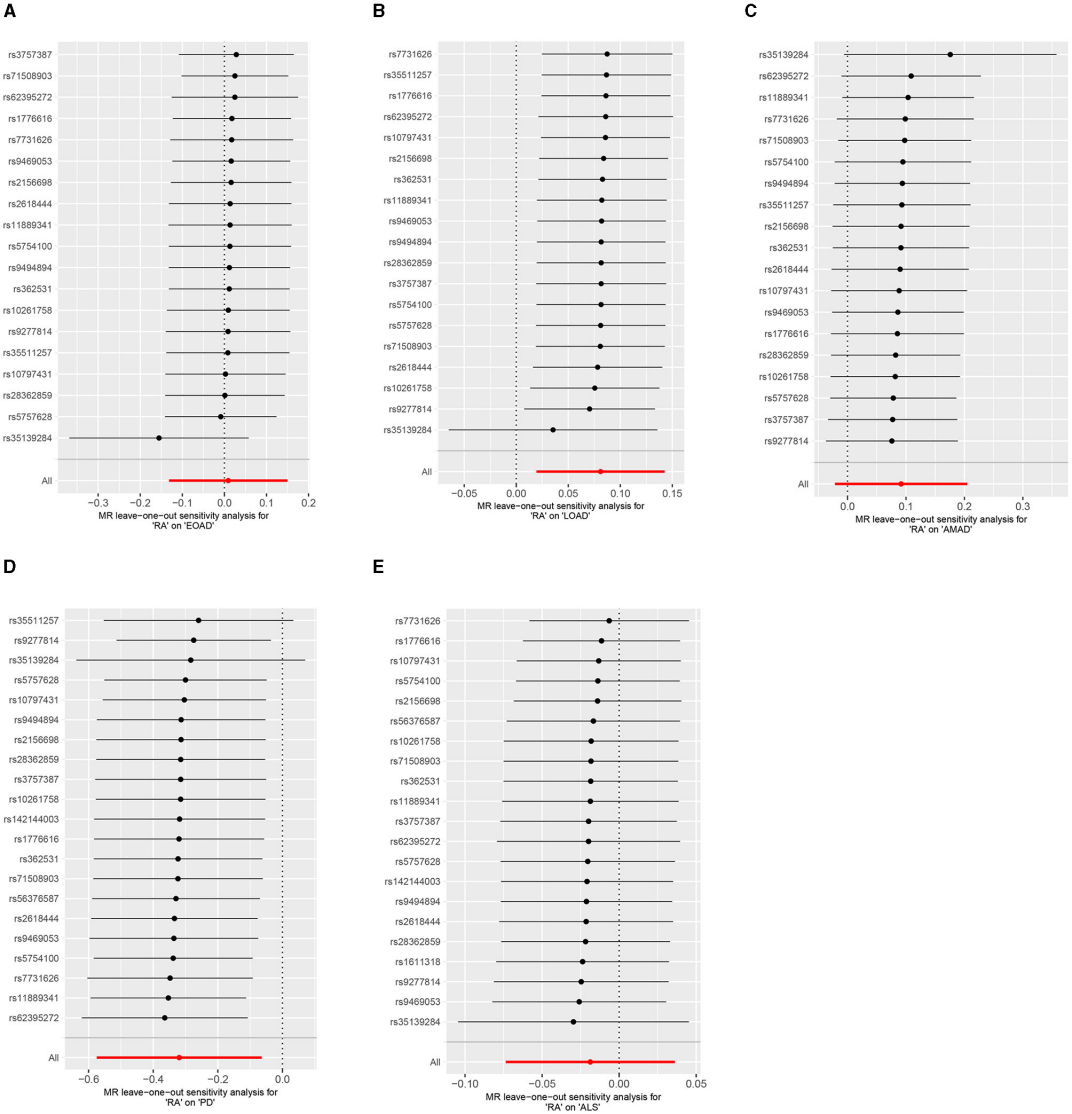
Leave-one-out analysis of RA on AD, PD, and ALS. **(A)** Early-onset Alzheimer's disease; **(B)** Late-onset Alzheimer's disease; **(C)** Atypical or mixed Alzheimer's disease; **(D)** Parkinson's disease; **(E)** Amyotrophic lateral sclerosis.

**Figure 6:**
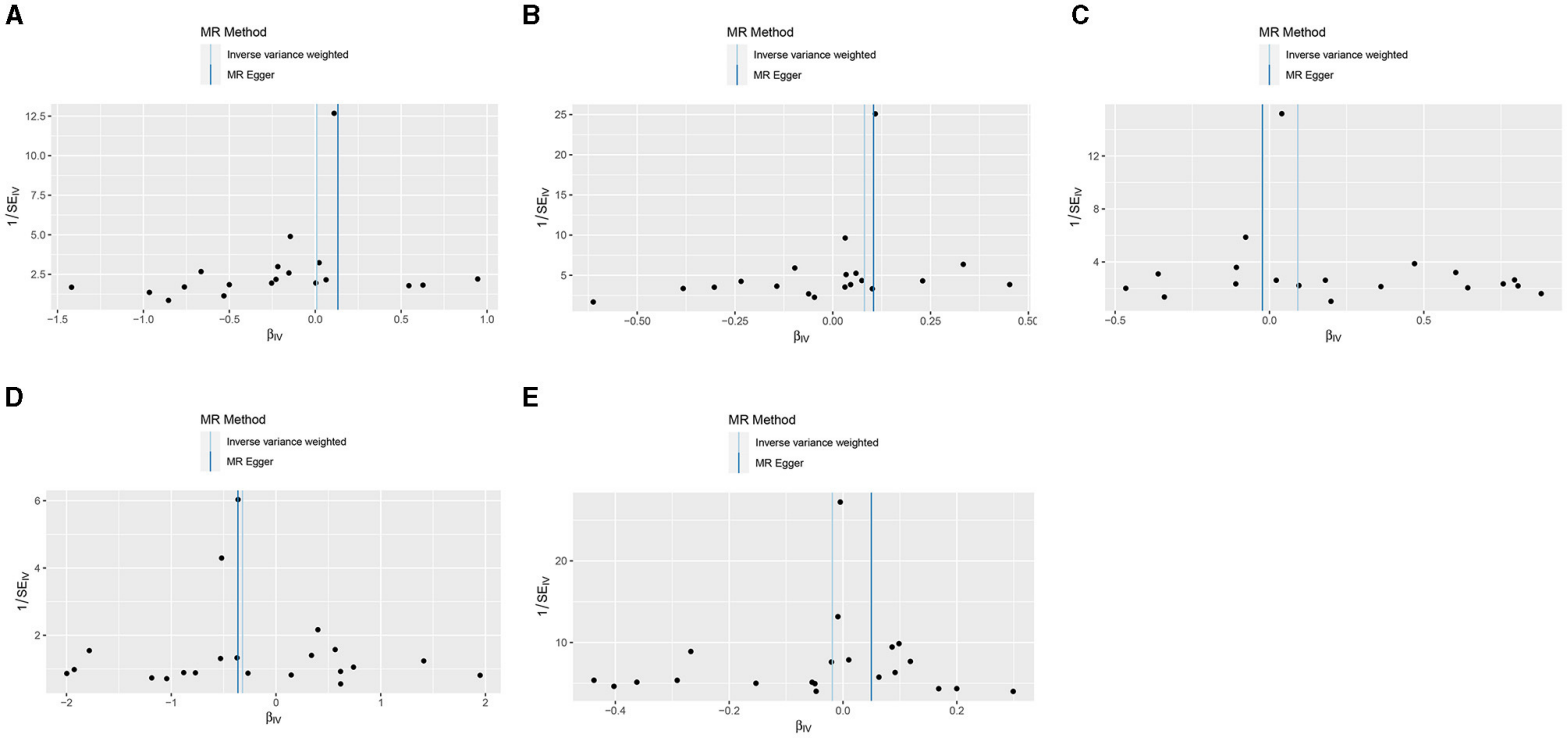
Funnel plots of the results from MR analysis of RA on AD, PD, and ALS. **(A)** Early-onset Alzheimer's disease; **(B)** Late-onset Alzheimer's disease; **(C)** Atypical or mixed Alzheimer's disease; **(D)** Parkinson's disease; **(E)** Amyotrophic lateral sclerosis.

Results from the MR sensitivity assessment was detailed in Tables 2, 3. The employment of heterogeneity tests, leveraging Cochrane's Q statistics, resulted in *p*-values surpassing 0.05. This suggests a lack of heterogeneity among the genetic variants analyzed. Moreover, the intercept obtained from the MR-Egger regression analysis, a tool designed to assess the potential for horizontal pleiotropy, did not reveal significant evidence of pleiotropy. This conclusion of no significant horizontal pleiotropy received further support from the results of the MR-PRESSO analysis. The leave-one-out sensitivity test identified the genetic variant rs35139284 as having a potential impact on the statistical relevance concerning LOAD and PD, whereas rs35511257 seemed to influence the significance of the findings related to PD. The detailed findings from the leave-one-out sensitivity test are depicted in Figure 5. Moreover, the funnel plots, as depicted in Figure 6, did not demonstrate any noticeable bias, thereby reinforcing the credibility and robustness of our research outcomes.

**Table 3:** Sensitivity analysis of the MR analysis results of rheumatoid arthritis with neurodegenerative diseases.

**Exposures**	**Outcomes**	**Heterogeneity test**		**Pleiotropy test**		**MR-PRESSO**
		**Cochran's Q test**	***p***	**Egger intercept**	***p***	**Global test (** ***p*** **)**
Rheumatoid arthritis	AD (EO)	24.122	0.151	−0.031	0.139	0.197
	AD (LO)	15.236	0.646	−0.006	0.525	0.627
	AD (AM)	22.395	0.215	0.029	0.082	0.269
	PD	27.196	0.130	0.013	0.758	0.185
	ALS	26.372	0.154	−0.014	0.063	0.203

AD, Alzheimer's disease; EO, Early onset; LO, Late onset; AM, Atypical or mixed; PD, Parkinson's disease; ALS, Amyotrophic lateral sclerosis.

## 4 Discussion

Utilizing a substantial volume of publicly accessible genetic information, our research delved into the causal associations between RA and three NDs. Through UVMR analysis, our study established a significant increase in the risk of LOAD due to RA, a finding that contradicts the MR results of Li et al. (46). Further MVMR analysis suggested that RA remained a risk factor for LOAD only after adjustment for alcohol drinking. However, when adjustments were made for BMI or T2DM, no association between RA and LOAD was observed. Moreover, our findings also revealed that RA may be a protective factor for PD, aligning with previous research conclusions (47). Nonetheless, this potential protective correlation was not deemed significant after making adjustments for BMI, alcohol drinking, or T2DM.

AD is categorized into EOAD and LOAD, distinguished by an age threshold of 65 years, with EOAD comprising about 4–6% of all AD instances (48). A multitude of preclinical investigations, systematic reviews, and meta-analyses have underscored the role of RA in the etiology of LOAD. Recent experimental findings demonstrated that inducing arthritis in APP/PS1 mice (a widely recognized model for AD) resulted in increased glial activation and aggravated amyloid deposition (49). Furthermore, a broad-based cohort study indicated that individuals with RA have a higher prevalence of AD and other dementia-related conditions compared to the general population (20). This observation was corroborated by a separate nested case-control study involving over 8.5 million adults, which confirmed the disparity of AD incidence across both young adults (mean age 42.1 years) and the elderly (65 years and older) (50). Actually, the existence of any inflammatory joint disease, particularly RA, is strongly correlated with later-life AD-related cognitive decline (21). Neuropsychiatric symptoms are also more common among RA patients (59.5%) than in their age-similar counterparts (17.1%) (51). A recent comprehensive analysis reiterated these results, revealing that patients with RA demonstrate markedly reduced performance in areas of attention, memory, and verbal abilities compared to controls matched for age (52). The aggregation of these data, in conjunction with our findings regarding the positive correlation between RA and LOAD, suggests a potential temporal link between chronic inflammation in RA and the initiation and worsening of cognitive impairment in AD. These findings facilitate the identification of RA patients with heightened susceptibility to LOAD, thereby enhancing monitoring and early intervention strategies to mitigate their risk. Additionally, clinicians can consider more proactive RA management approaches for RA patients, particularly those with a familial history of LOAD, to further diminish the likelihood of developing LOAD.

The underlying mechanisms by which RA increases the risk of LOAD are currently unclear. AD is pathologically typified by the extracellular build-up of amyloid-β plaques and the intracellular aggregation of tau neurofibrillary tangles, both of which result in a gradual, time-dependent neuronal degradation and consequent functional loss (4). Some theories propose a connection between systemic inflammatory disorders and neuroinflammation, attributable to common biological mechanisms. RA exemplifies such an autoimmune condition with elevated inflammatory activity. In RA patients, specific biomarkers become detectable in the serum, including amyloid A protein, anti-cyclic citrullinated peptide, rheumatoid factor, C-reactive protein and calgranulin (53). The existence of amyloid structures is particularly intriguing, given that light chain amyloidosis of transthyretin and immunoglobulins leads to amyloid deposition in soft tissues (54). Furthermore, chronic systemic peripheral inflammation impacts the neurodegenerative processes inherent in AD. Inflammatory cytokines such as interleukin-6 (IL-6), interleukin-1beta (IL-1β), tumor necrosis factor-alpha (TNF-α), interleukin-12 (IL-12), and interleukin-18 (IL-18), and transforming growth factor beta (TGF-β) exhibit increased activity in AD patients relative to healthy individuals (55). Researchers are studying these cytokines and their effects in the pathogenesis of both AD and RA, given that an overactive immune response is a commonality in these conditions. It is worth mentioning that the blood-brain barrier (BBB) serves as a mediator between RA and AD. Empirical research suggested that RA patients exhibit altered BBB permeability. BBB dysfunction is also linked to NDs, including AD (56). However, our analysis indicated no association between RA and LOAD when adjusted for BMI or T2DM, suggesting that BMI and T2DM might be confounding factors influencing RA and LOAD. Obesity is a known proponent of systemic inflammation, which can precipitate insulin resistance, β-cell dysfunction, and eventually T2DM, with these conditions being implicated in the pathophysiology of both AD and RA (57). In light of these findings, the intricate physiological interactions between RA and AD warrant further investigation to elucidate their potential mechanistic links.

While our research indicated that individuals with RA exhibit a lower likelihood of developing PD, the precise reasons behind this protective influence remain largely undetermined. This observation stands in contrast to earlier theories suggesting that sustained inflammation and an excess of pro-inflammatory molecules in autoimmune conditions could potentiate microglial activation and neuronal degeneration, potentially heightening PD susceptibility (19, 58). Notably, an increasing body of research highlights the significance of lysosomal malfunction in both autoimmune diseases and NDs (59). Specifically, in PD, diminished activity of lysosomal enzymes can result in the buildup of α-synuclein and the creation of Lewy bodies, a critical pathological feature of PD (60, 61). Furthermore, the study has demonstrated that heightened expression of lysosomal proteases, such as cathepsin D, can mitigate α-synuclein aggregate formation in murine models (62). Remarkably, autoimmune conditions, including RA, often exhibit increased lysosomal enzyme activities (59, 63). This is evidenced by elevated concentrations of various lysosomal cathepsins in the serum and synovial fluid of RA sufferers, a stark contrast to the reduced enzyme activity seen in PD (64). Thus, the lysosome pathway may offer a protective mechanism against PD in RA patients, meriting further investigation. Nonetheless, our MVMR analysis found no significant association between RA and PD once adjustments were made for BMI, alcohol drinking, or T2DM. Obesity has been reported to be associated with lysosomal dysfunction. Excess body weight might lead to the increase of intracellular lysosome burden and affect its normal function. Additionally, obesity is frequently accompanied by chronic low-grade inflammation of adipose tissue, a condition that may alter lysosomal activity and protein expression (65). Moreover, long-term excessive drinking can damage liver cells and impair lysosomal function. Harmful substances (such as acetaldehyde) produced in the process of alcohol metabolism may damage lysosomal membrane and affect its normal function (66, 67). Insulin resistance, a hallmark of T2DM, has been linked to lysosomal dysfunction, with the study suggesting that lysosomes play a pivotal role in insulin signaling and glucose metabolism (68). Therefore, it can be speculated that the connection between obesity, alcohol intake, and T2DM with lysosomal dysfunction could obscure the potential link between RA and PD. Furthermore, robust epidemiological data have consistently shown that T2DM augments both the risk and progression rate of PD (69). Additionally, T2DM prevalence is notably higher in RA patients compared to healthy counterparts (70). There is a well-documented association between obesity and PD, with evidence pointing to a relationship between increased BMI, systemic inflammation, and the severity of PD (71–74). Recent findings also indicated that individuals with RA have a higher incidence of low lean mass and sarcopenic obesity compared to the general population (75). Lifestyle habits, such as alcohol use, could also influence PD development. It was reported that compared to moderate drinkers, abstainers and heavy drinkers face a heightened risk of developing Hoehn and Yahr stage 3 PD (76). Given that RA patients are more susceptible to obesity and T2DM, and may alter their alcohol consumption following an RA diagnosis, these factors could partially obscure RA's direct protective impact on PD. It is imperative to conduct further research into the complex interplay of factors influencing the relationship between RA and PD. Should further studies validate the protective impact of RA on PD, it would enable the development of more precise treatment and prevention strategies, tailored to the patient's genetic background and disease characteristics.

Our study is the first to explore the causal relationship between RA and ALS. Both UVMR and MVMR results suggested no causal associations between RA and ALS. The origins of ALS remain largely enigmatic, with ongoing debate about whether autoimmune processes contribute to its development or if there is a connection with inflammatory or autoimmune conditions such as RA. Reports of RA patients developing ALS are scarce, and epidemiological studies that investigate the simultaneous occurrence of these conditions are limited (77–79). Prior research has determined that the incidence of ALS among individuals with RA aligns with that observed in the general population, once adjusted for age and gender, suggesting that ALS and RA are likely distinct conditions with minimal, if any, shared etiological factors (80). To further clarify the relationship between RA and ALS onset, as well as to uncover the mechanisms underlying this relationship, extensive cohort studies are required.

The present study is not without its limitations. First, the GWAS summary statistics utilized were derived exclusively from European cohorts, which poses questions about the generalizability of our results to ethnically diverse groups. Further research is needed to compare the findings from this European cohort with those from other ethnic groups, such as East Asians, to elucidate the global association between RA and NDs. Second, the GWAS dataset for PD employed in MR analysis comprised a relatively small number of PD cases, somewhat constraining the reliability and robustness of the findings. Further research is needed to validate these results in a larger cohort of PD patients. Third, our exploration of the association between RA and specific subtypes of PD or ALS was constrained by the paucity of SNP data in the existing database. Fourth, akin to all MR analyses, our methodology cannot completely dismiss the influence of latent pleiotropy, which may skew our findings. This underlines the necessity for more comprehensive research to elucidate the connections between RA and NDs.

## 5 Conclusion

In summary, our analyses utilizing UVMR and MVMR with adjustment for alcohol drinking provide evidence supporting a significant causal effect of RA on elevating the risk of LOAD, thereby identifying RA as a potential risk factor for LOAD. Simultaneously, our UVMR analysis suggested a potential inverse correlation between RA and PD, suggesting a potential protective role of RA against PD. It is of paramount importance to validate these results with extensive prospective research and to explore the biological mechanisms underlying these relationships.

## Data availability statement

The original contributions presented in the study are included in the article/Supplementary material, further inquiries can be directed to the corresponding author.

## Author contributions

XC: Formal analysis, Methodology, Data curation, Investigation, Visualization, Writing—original draft. LC: Conceptualization, Methodology, Supervision, Writing—review & editing. WF: Formal analysis, Investigation, Writing—review & editing. QY: Data curation, Investigation, Writing—review & editing. XM: Investigation, Methodology, Writing—review & editing. LY: Data curation, Formal analysis, Methodology, Project administration, Software, Visualization, Writing—original draft.
